# Thermoelectric transport in coupled double layers with interlayer excitons and exciton condensation

**DOI:** 10.1103/PhysRevB.102.235304

**Published:** 2020-12-29

**Authors:** Jiuning Hu, Albert F. Rigosi, David B. Newell, Yong P. Chen

**Affiliations:** 1 National Institute of Standards and Technology, Gaithersburg, Maryland 20899, USA; 2 School of Electrical and Computer Engineering, Purdue University, West Lafayette, Indiana 47907, USA; 3 Birck Nanotechnology Center, Purdue University, West Lafayette, Indiana 47907, USA; 4 Department of Physics and Astronomy, Purdue University, West Lafayette, Indiana 47907, USA

## Abstract

Quantum Boltzmann formalism is employed to study the transport properties of strongly-coupled double layer systems that enable the formation of interlayer excitons and exciton condensation. The importance of exciton formation, dissociation, and condensation is highlighted in the context of thermoelectric power generation, and this mathematical inquiry provides an alternative methodology to calculate the thermoelectric efficiency given the conditions of exciton formation. The Onsager relation for the Coulomb drag resistivity is shown to be valid even when exciton condensation is present. In addition, it is found that the traditional thermoelectric figure of merit is no longer sufficient to predict the efficiency of thermoelectric power generation in the presented situations. This inquiry offers insights for designing double layer systems, including their interlayer interactions, with enhanced thermoelectric energy conversion efficiency.

## I. INTRODUCTION

Thermoelectric generators and Peltier refrigerators with high efficiencies have been actively pursued for decades. To achieve comparable efficiencies with dynamic heat engines and refrigerators, the dimensionless thermoelectric figure of merit $ZT=\frac{\sigma \alpha^{2}T}{\kappa }$ for thermoelectric materials needs to be approximately 4 [1], where *σ* is electrical conductivity, *α* is the Seebeck coefficient, *κ* is thermal conductivity, and *T* is temperature. Various approaches to optimize thermoelectric material structures and dimensionality to enhance the *ZT* value have been pursued in the past. For example, “electron-crystal-phonon-glass” systems have generated great interest [2,3]. Despite tremendous effort, experimental *ZT* values are still notably below 4, not mentioning the complications in applications due to costly materials or high temperatures [1,4]. Seeking alternative high *ZT* materials and devices continues to be a vigorous research field.

The traditional thermoelectric module usually consists of two spatially separated channel materials of opposite doping in which the Coulomb interaction between them is negligibly small. However, the Coulomb interaction can be made strong if the channels are very thin and brought into close proximity, and in this case, the thermoelectric performance may be significantly enhanced. Stacked two-dimensional (2D) systems are ideal candidates for studying such thermoelectric transport with strong interchannel Coulomb interactions.

Two-dimensional materials and heterostructures offer promising opportunities to take advantage of the relevant physics via device engineering. Applications range from building high-mobility systems for quantum computation by enabling the existence of anyons [5] to Bose-Einstein condensation (BEC) of excitons with excitonic superfluidity [6-9]. Ever since the first isolation of graphene [10], various 2D materials have likewise been isolated from their bulk counterparts and can be grown on substrates. In addition, 2D materials such as graphene, hexagonal boron nitride (hBN), and transition metal dichalcogenides, can be stacked [11-15] to form high-quality interfaces, having both tunable interlayer distances and interactions [16].

In this paper, we report a mathematical approach to understanding transport properties in coupled double layer systems and show how the formation of excitons and exciton condensation affect the transport coefficients. The important role of exciton formation, dissociation, and condensation in deriving the formula for thermoelectric efficiency is established. Since the traditional *ZT* value is not sufficient to understand thermoelectric efficiency in the presence of exciton or exciton condensation, this approach offers new insights relevant to designing high efficiency modules.

Foundational principles and quantum Boltzmann formalism for unbounded carriers and excitons are described in Sec. II, wherein the formula for transport coefficients are provided and the Onsager relation is proved. In Sec. III, exciton condensation and its interaction with noncondensed excitons is discussed based on Zaremba-Nikuni-Griffin formalism [17,18], which has been successfully applied to BECs at finite temperatures. Section IV discusses thermoelectric applications and quantitative insights for designing high *ZT* modules in the presence of excitons or exciton condensation.

## II. BOLTZMANN TRANSPORT FORMALISM IN COUPLED DOUBLE LAYER SYSTEMS

Before introducing transport formalism, it will benefit the reader to establish some foundational principles, starting with a discussion of the resistivity matrix and how one incorporates the strength of interlayer interactions. For a Coulomb-coupled double layer system, the effect of the interlayer interaction can be included in the Coulomb drag resistivity, whose tensor may be reduced to a matrix for anisotropic one-dimensional transport in each layer:

(1) $$\rho =\left[\begin{array}{cc} \rho_{t} & \rho_{D}\\ \rho_{D} & \rho_{b} \end{array}\right],$$

where *ρ*_*t*(*b*)_ is the resistivity for the layer with label *t*(*b*) and *ρ_D_* is the Coulomb drag resistivity. The entropy production is *σ_R_* = **I**^*T*^ *ρ***I**, where **I** = [*I_t_ I_b_*]^*T*^ is the vector of prescribed current flowing in both layers (the superscript ^*T*^ is for the vector transpose). Since *σ_R_* should be non-negative (*ρ* is positive semidefinite) for any prescribed currents, it follows that:

(2) $$-\sqrt{\rho_{t}\rho_{b}}\leq \rho_{D}\leq \sqrt{\rho_{t}\rho_{b}}.$$

Therefore, the magnitude of Coulomb drag resistivity is bounded by the geometric mean of the intralayer resistivities. If the equality in Eq. (2) holds, i.e., $\rho_{D}=\pm \sqrt{\rho_{t}\rho_{b}}$, the entropy production will be zero if $\sqrt{\rho_{t}}I_{b}=\mp \sqrt{\rho_{b}}I_{t}$. Thus, dissipationless current can flow in coupled double layers when the resistivity matrix is singular. If one layer is superconducting, e.g., *ρ_t_* = 0, we will have *ρ_D_* = 0, consistent with reported experiments [19-22].

**FIG. 1.**
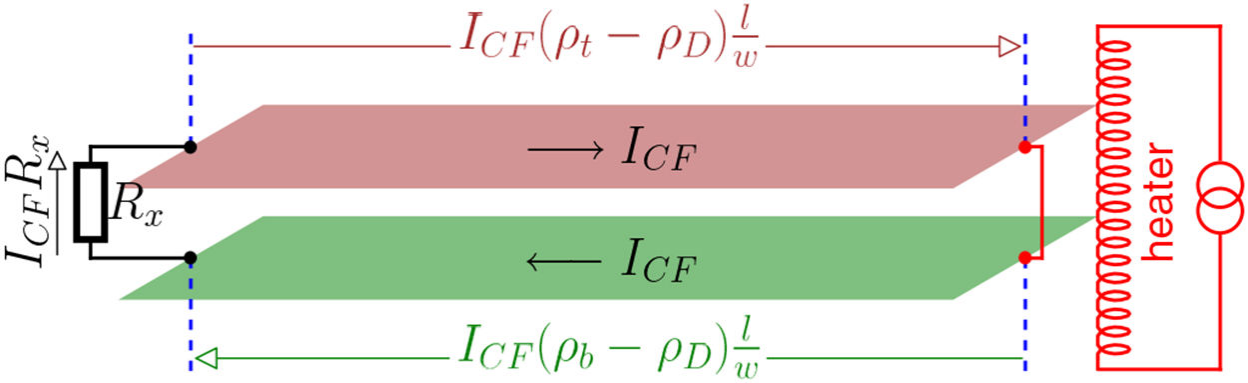
Counterflow thermoelectric transport for a generic double layer system with interlayer interactions. The formulas on closed-headed arrows —> represent the voltage drop along the arrows between the indicated ends. The open-headed arrows → represent the current direction, with *l* and *w* being the length (along current direction) and width (transverse to current direction) of each layer, respectively.

Now consider the counterflow thermoelectric circuit represented by the schematic in Fig. 1. It consists of the building blocks of traditional thermoelectric modules. The two layers (red and green) are shorted at the right side, and a resistance *R_x_* is connected between the two layers at the left side. Here *R_x_* includes a load resistance and any extrinsic resistances of the contacts and interconnects. Assuming that the counterflow Seebeck coefficient is *α_CF_*, the voltage is measured between the two red dots in Fig. 1 after the red wire is removed and a temperature difference of 1 K is established between the black and red dots. A heater at the right side will create a temperature difference Δ*T* between the two ends of each layer that induces an electromotive force *α_CF_* Δ*T* to drive the counterflow current *I_CF_*. From Fig. 1, we have

(3) $$I_{CF}=\frac{\alpha_{CF}\Delta T}{\left[R_{x}+(\rho_{t}+\rho_{b}-2\rho_{D})\frac{1}{w}\right]}.$$

The effective electrical conductivity from Eq. (3) is

(4) $$\sigma_{\text{eff}}=\frac{l}{wR_{x}+(\rho_{t}+\rho_{b}-2\rho_{D})l}.$$

If the counterflow transport is dissipationless, i.e., *ρ_D_* = *ρ_t_* = *ρ_b_*, *I_CF_* = *α_CF_* Δ*T*/*R_x_* and *σ*_eff_ = *l*/(*wR_x_*). The thermoelectric figure of merit for such a dissipationless system (connected by the external resistor *R_x_*) would then become:

(5) $$ZT=\frac{\alpha_{CF}^{2}T}{\kappa wR_{x}}l.$$

There are two important things to note here. The first is that the units of ZT are still what they would normally be for traditional cases since a length unit exists in both the numerator and denominator (as length and width, respectively). The second is that we do not consider the dimensions of the two layers when discussing thermal resistance because we do not have an extrinsic thermal resistor incorporated into the circuit (whereas the discussion of a total electrical current and external resistor necessitated the inclusion of the layers’ lateral dimensions). It is exclusively the existence of an external resistor, coupled with a superconducting top and bottom layer, that renders ZT proportional to the length *l* of the thermoelectric unit [23]. Thus, double layer systems with any Coulomb drag resistivity may have a high *ZT* by simply optimizing the system geometry.

**FIG. 2.**
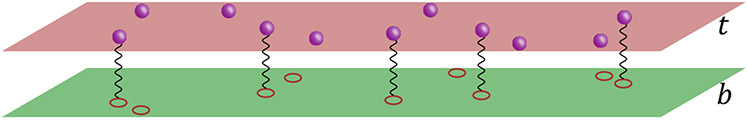
Illustration of a double layer system. The layer indices are at the right end of each layer. Excitons are composed of two carriers, represented by circles, and are sinusoidally connected across the two layers. The other circles without connections represent the unbounded carriers.

Though the phenomenological model is inadequate for giving a full picture of thermoelectric transport in coupled double layer systems, it does offer several insights including the idea that large Coulomb drag resistivity, due to strong interlayer interactions, can result in high *ZT* values. In general, to have positive *ρ_D_*, the two layers should have charges of opposite sign. For instance, Coulomb interactions between electrons in one layer and holes in the other may enable the formation of interlayer excitons, as drawn in Fig. 2.

Another component that will be considered in this formalism is exciton BECs, which are predicted to occur in double layer systems potentially near room temperature, although different theories have differed substantially on the critical temperature for the condensation [24-33]. At present, condensation has only been observed at low temperature and high magnetic field [6-9]. That said, efforts are ongoing pertaining to both condensates and double layer systems [34-41]. While it is proposed that the formation of excitons can lead to high *ZT* [23,42], to date there have been no systematic studies on thermoelectric transport in coupled double layers, such as the one depicted in Fig. 2, with interlayer exciton condensation. Counterflow Seebeck coefficients and power factors have been measured in bilayer graphene, a double layer system with Coulomb drag, but no signature of interlayer excitons or exciton condensation was observed [43].

At finite temperature, the exciton condensation fraction is less than 100%, and thus the system in Fig. 2 can support the coexistence of exciton condensate, noncondensed excitons, and unbounded carriers. It has been shown that Boltzmann formalism and Bose superfluid hydrodynamics can successfully be used to describe both noncondensates and condensates for dilute Bose gases [17,18,44]. We consider the case where the interexciton distance is larger than the interlayer distance, so that excitons can be well approximated by bosons and the Boltzmann formalism for dilute Bose gases can be applied to excitons. We also assume that the formation of excitons occurs at a temperature higher than the exciton *condensation* temperature, which corresponds to the BEC limit [45]. In such a scenario, the interaction between the exciton condensate and noncondensate will help to determine the condensate velocity when the noncondensate and unbounded carriers are driven out of equilibrium by external fields or a temperature gradient. If the unbounded carriers are neglected, then only counterflow electrical current is allowed in two layers. It is therefore necessary to allow unbounded carriers entering the formalism to have noncounterflow transport. Additionally, such unbounded carriers must exist when the carrier densities before exciton formation in both layers are different.

Though, in classical cases, ZT does not scale with the dimension of the system, it should be noted that the opposite is true in this case because of the emergence of superfluidity. In the cases presented in this work, the existence of exciton condensation allows electrical and thermal transport to scale differently, removing the apparent inconsistency of why the classical case does not apply. Furthermore, with the existence of an exciton condensation, electrical current is carried by condensation, which itself exhibits superfluidity, whereas the heat current is carried by excitations. This distinction is crucial in our explanations for why traditional expectations of ZT cannot be applied.

To begin the formalism, the properties of the double layer system in Fig. 2 can be encoded in the distribution functions *f_μ_*(**x, k**_*μ*_, *τ*), where *μ* = *t, b* for the unbounded carriers in the layer *t, b* and *μ* = *e* for the noncondensate excitons. The dynamics of *f*_*μ*_ can be described by the quantum Boltzmann equations that have been successfully used to study the interactions between BEC and its excitations [17,18,44]. Here **x** is the 2D position of particles in each layer, **k**_*μ*_ is the wave vector, and *τ* is time. The generic coupled Boltzmann equations for the distribution functions are [46]

(6) $$\partial_{\tau }f_{\mu }+v_{\mu }\cdot \nabla_{x}f_{\mu }+\frac{F_{\mu }}{\hbar }\cdot \nabla_{k}f_{\mu }=C_{\mu }[f],$$

where *f* represents all the distribution functions *f_t_*, *f_b_*, and *f_e_*. The collision integral contains components from single particle scattering $\left(C_{\mu }^{s}\right)$, binary scattering $\left(C_{\mu }^{b}\right)$, exciton formation and decay $\left(C_{\mu }^{r}\right)$, and scattering of noncondensate excitons by exciton condensate $\left(C_{\mu }^{c}\right)$, i.e.,

(7) $$C_{\mu }[f]=C_{\mu }^{s}[f]+C_{\mu }^{b}[f]+C_{\mu }^{r}[f]+C_{\mu }^{c}[f].$$

More details of these integrals, and the application of external fields, can be found in Appendix A. The exciton condensate, if it exists, enters Eq. (6) through the collision integral $C_{\mu }^{c}$. While we are mainly interested in the steady state transport properties, exciton condensates may be considered a static background, with its governing equations discussed in a later section.

### A. Linearized Boltzmann equations

Assuming a uniform electric field in each layer, the explicit position dependence in both *f*_*μ*_ and the transition probabilities may be dropped despite many quantities, such as carrier concentration, still being position dependent through local variables like temperature. Furthermore, it is assumed that only electric fields and temperature gradients are the only major factors that drive the distribution out of equilibrium. Therefore, the derivation of *f*_*μ*_ from the local equilibrium distribution function

(8) $$n_{\mu }(x,k_{\mu })={\left[\text{exp}\left(\frac{e_{\mu }(k_{\mu })-\zeta_{\mu }(x)}{k_{B}T(x)}\right)+(-1{)}^{\delta_{\mu e}}\right]}^{-1}$$

is proportional to the electric fields and temperature gradients, where *k_B_* is the Boltzmann constant, *T*(**x**) is the temperature, and *ζ*_*μ*_(**x**) is the chemical potential. Linear response theory can then be applied to calculate the transport coefficients. We can write

(9) $$f_{\mu }=n_{\mu }(1-{\bar{n}}_{\mu }\phi_{\mu })$$

where ${\bar{n}}_{\mu }=1-(-1{)}^{\delta_{\mu e}}n_{\mu }$. Now the linearized steady state Boltzmann equations (see Appendix B) can be written as

(10) $$X_{\mu }\equiv \frac{1}{\hbar }\left(F_{\mu }-\frac{e_{\mu }-\zeta_{\mu }}{T}\nabla_{x}T\right)\cdot \nabla_{k}n_{\mu }=\sum_{v}P_{\mu ,v}\phi_{v},$$

where $q_{\mu }E_{\mu }=F_{\mu }\equiv F_{\mu }-\nabla_{x}\zeta_{\mu }$ is the gradient of the electrochemical potential (at local chemical equilibrium *ζ_e_* = *ζ_t_* + *ζ_b_* and thus $F_{e}=F_{t}+F_{b}$). As stated earlier, the **x** dependence of the transition probabilities *T* and *ζ*_*μ*_ will be removed.

### B. Transport coefficients

It can be shown that P is a positive-definite operator, thus the inverse of P exists and it is denoted by $P^{-1}=L$. The solution of the Boltzmann equations can be written as

(11) $$\phi_{\mu }=\sum_{v=t,b,e}L_{\mu ,v}X_{v}.$$

Note that such a solution is formal for any generic collision integrals given in Appendix B. An explicit formula for L will not be given here since it can only be solved numerically, except for some cases involving simple approximations (such as the relaxation time approximation). Attention should be paid to the approximated solutions when symmetry properties of L are demanded, as for the Onsager reciprocal relations shown in the next subsection. There are already numerous efforts devoted to various numerical methods [47,48] that should be consulted when a particular system is considered.

The normal electric current in each layer carried by the unbounded carriers and noncondensate excitons (see Appendix C) is

(12) $$j_{\mu }^{n}=\sum_{v=t,b}\sigma_{\mu ,v}E_{v}-\tau_{\mu }\nabla_{x}T,$$

where the coefficients’ matrices can be formally written as

(13) $$\sigma_{\mu ,v}=\frac{k_{B}Tq_{\mu }q_{v}}{\hbar^{2}}(J_{\mu ,v}+J_{\mu ,e}+J_{e,v}+J_{e,e}),\;\;\tau_{\mu }=\frac{k_{B}q_{\mu }}{\hbar^{2}}(K_{\mu ,t}+K_{\mu ,b}+K_{\mu ,e}+K_{e,t}+K_{e,b}+K_{e,e}),$$

with

(14) $$J_{\mu ,v}\equiv \int \nabla_{k}n_{\mu }⊗L_{\mu ,v}\nabla_{k}n_{v}dk_{\mu },\;K_{\mu ,v}\equiv \int \nabla_{k}n_{\mu }⊗L_{\mu ,v}[(e_{v}-\zeta_{v})\nabla_{k}n_{v}]dk_{\mu },$$

in which the notation (**a** ⊗ **b**)_*mn*_ ≡ *a_m_b_n_* has been used.

Similarly, the heat current (see Appendix C) in each layer (*μ* = *t, b*) can be written as

(15) $$h_{\mu }=\sum_{v=t,b}\chi_{\mu ,v}F_{v}-\kappa_{\mu }\nabla_{x}T,$$

where the coefficients’ matrices are

(16) $$\chi_{\mu ,v}=\frac{k_{B}T}{\hbar^{2}}\left(M_{\mu ,v}+M_{\mu ,e}+\frac{1}{2}M_{e,v}+\frac{1}{2}M_{e,e}\right),\;\kappa_{\mu }=\frac{k_{B}}{\hbar^{2}}\left(N_{\mu ,t}+N_{\mu ,b}+N_{\mu ,e}+\frac{1}{2}N_{e,t}+\frac{1}{2}N_{e,b}+\frac{1}{2}N_{e,e}\right),$$

with

(17) $$M_{\mu ,v}\equiv \int (e_{\mu }-\zeta_{\mu })\nabla_{k}n_{\mu }⊗L_{\mu ,v}\nabla_{k}n_{v}dk_{\mu },\;N_{\mu ,v}\equiv \int (e_{\mu }-\zeta_{\mu })\nabla_{k}n_{\mu }⊗L_{\mu ,v}[(e_{v}-\zeta_{v})\nabla_{k}n_{v}]dk_{\mu }.$$

Note that the factor $\frac{1}{2}$ appears in Eq. (16) because one exciton contributes equally to the heat currents **h**_*t*_ and **h**_*b*_.

For an open circuit, $j_{\mu }^{n}=0$, and thus a temperature gradient will induce intralayer electric fields. The Seebeck coefficient can be expressed as

(18) $$\alpha_{\mu }=\sum_{v=t,b}\rho_{\mu ,v}\tau_{v},$$

where *ρ_μ,ν_* is the inverse of *σ_μ,ν_* such that ∑*_ν=t,b_σ_μ,ν_ρ_ν,ω_* = *δ_μω_I* where *I* is the unity matrix. Note that such an inverse will always exist because of the fact that P is positive definite.

### C. Onsager reciprocal relations

Before studying the symmetry properties of the conductivity matrix, we will examine the operator P. The most generic collision integral is given in Appendix B and the generic term $C_{\mu }^{g}[\phi ](k_{\mu })$ in *C*_*μ*_[*f*] after linearization can be written as

(19) $$\sum_{v}P_{\mu ,v}^{g}\phi_{v}=Q^{a}(k_{\mu })\phi_{\mu }+\sum_{v}\int Q^{b}(k_{\mu };k_{v})\phi_{v}dk_{v}.$$

It can be verified that

(20) $$\langle \psi_{\mu },P_{\mu ,v}^{g}\phi_{v}\rangle -\langle \phi_{v},P_{v,\mu }^{g}\psi_{\mu }\rangle \;\;=\int [Q^{b}(k_{\mu };k_{v})-Q^{b}(k_{v};k_{\mu })]\psi_{\mu }\phi_{v}dk_{\mu }dk_{v},$$

where the inner product is defined as ⟨*ψ*_*μ*_, *ϕ*_*μ*_⟩ ≡ *∫ ψ_μ_ϕ_μ_*d**k**_*μ*_. Therefore $P_{\mu ,v}^{g}$ will be self-adjoint if

(21) $$Q^{b}(k_{\mu };k_{v})=Q^{b}(k_{v};k_{\mu }),$$

that is, *Q^b^* is symmetric with respect to its arguments. The existence of such symmetry is dictated by the detailed balance of the scattering processes, as shown in Appendix B for *Q*_1/2/3/*c*_. On the other hand, the charge conservation for each individual layer (no tunneling current) requires $\sum_{v=\mu ,e}\int C_{v}^{g}[\phi ](k_{v})dk_{v}=0$ which is then equivalent to

(22) $$(\delta_{\alpha \mu }+\delta_{\alpha e})Q^{a}(k_{\alpha })+\sum_{v=\mu ,e}\int Q^{b}(k_{v};k_{\alpha })dk_{v}=0.$$

By performing summation over *μ* = *t, b* for Eq. (22), *Q^a^* and *Q^b^* are connected by

(23) $$Q^{a}(k_{\mu })=-\sum_{v=t,b,e}\frac{1+\delta_{ve}}{1+\delta_{\mu e}}\int Q^{b}(k_{v};k_{\mu })dk_{v},$$

for *μ* = *t, b, e*. We then have

(24) $$\sum_{\mu =t,b,e}\langle \phi_{\mu },C_{\mu }^{g}[\phi ]\rangle \;\;=-\frac{1}{2}\sum_{\mu ,v=t,b,e}\int Q^{b}(k_{\mu };k_{v})\lambda [\phi_{\mu },\phi_{v}{]}^{2}dk_{\mu }dk_{v},$$

where $\lambda [\phi_{\mu },\phi_{v}]\equiv \sqrt{\frac{1+\delta_{ve}}{1+\delta_{\mu e}}}\phi_{\mu }-\sqrt{\frac{1+\delta_{\mu e}}{1+\delta_{ve}}}\phi_{v}$ and Eq. (21) is used. It can be verified that Eqs. (21), (23), and (24) hold for all *C^s/b/r/c^* listed in Appendix B.

For each of the processes *C^s/b/r/c^* we considered, we have *Q^b^* (**k**_*μ*_; **k**_*ν*_) < 0. Therefore

(25) $$\sum_{\mu ,v=t,b,e}\langle \phi_{\mu },P_{\mu ,v}\phi_{v}\rangle =\sum_{\mu =t,b,e}\langle \phi_{\mu },C_{\mu }^{g}[\phi ]\rangle \geq 0,$$

and the equality holds only if *ϕ_μ_* = 0 for all *μ* = *t, b, e*. As a result, P is positive definite. Thus $L=P^{-1}$ must exist and is also self-adjoint and positive definite. We then arrive at the following Onsager relations

(26) $$\sigma_{\mu ,v}=\sigma_{v,\mu }^{T},\;\;\sum_{v=t,b}\chi_{v,\mu }=\frac{T}{q_{\mu }}\tau_{\mu }^{T},\;\;\sum_{\mu =t,b}\kappa_{\mu }=\sum_{\mu =t,b}\kappa_{\mu }^{T},$$

for *μ* = *t, b*, where the superscript ^*T*^ indicates a matrix transpose. The summation over individual layers appears since the temperature profile is identical for every layer, whereas the electric field in each layer can be independently tuned. The second equation of Eq. (26) highlights the symmetry between the responses of the electric and heat currents to ∇_**x**_*T* and the electrochemical force field $F_{t}=F_{b}$, respectively. For the third equation of Eq. (26), the Onsager relation might break down for individual layers, i.e., $\kappa_{\mu }\neq \kappa_{\mu }^{T}$.

It is the self-adjointness of all $P^{s∕b∕r∕c}$ that determines the validity of the Onsager reciprocal relations. If interlayer tunneling exists, or Eq. (23) is violated, the Onsager relations still hold provided L exists (a physical requirement). Any linearized collision integral that violates Eq. (21) will break down the Onsager relations and may be considered nonphysical.

### D. Entropy production

The entropy density and entropy current can be defined as [46]

(27) $$s_{\mu }\equiv -k_{B}\int [f_{\mu }\;\text{ln}\;f_{\mu }+(-1{)}^{\delta_{\mu e}}{\bar{f}}_{\mu }\;\text{ln}\;{\bar{f}}_{\mu }]dk_{\mu },\;u_{\mu }\equiv -k_{B}\int v_{\mu }[f_{\mu }\;\text{ln}\;f_{\mu }+(-1{)}^{\delta_{\mu e}}{\bar{f}}_{\mu }\;\text{ln}\;{\bar{f}}_{\mu }]dk_{\mu }.$$

It then can be shown that

(28) $$\partial_{\tau }s_{\mu }+\nabla_{x}\cdot u_{\mu }=\int \frac{e_{\mu }-\zeta_{\mu }}{T}C_{\mu }[f](k_{\mu })dk_{\mu }+k_{B}\langle \phi_{\mu },X_{\mu }\rangle .$$

Due to the energy and particle number conservation, the first term on the right hand side of Eq. (28) is zero. Note that *∫* (*e_μ_* − *ζ_μ_*)*C_μ_*[*f*](**k**_*μ*_)d**k**_*μ*_ = 0 for combined energy and particle number conservation for $C_{\mu }^{r}$ whereas *∫ e_μ_C_μ_*[*f*](**k**_*μ*_)d**k**_*μ*_ = *∫ ζ_μ_C_μ_*[*f*](**k**_*μ*_)d**k**_*μ*_ = 0 for $C_{\mu }^{s}$ and $C_{\mu }^{b}$. In the next section, we show that $\int (e_{e}-\zeta_{e})C_{e}^{c}[f](k_{e})dk_{e}$ is of third order with respect to the magnitude of the exciton condensate velocity and can thus be neglected. The total local entropy generation then becomes

(29) $$s=k_{B}\sum_{\mu =t,b,e}\langle \phi_{\mu },X_{\mu }\rangle =\sum_{\mu =t,b}j_{\mu }\cdot E_{\mu }+\sum_{\mu =t,b}h_{\mu }\cdot \nabla_{x}\left(\frac{1}{T}\right)\;\;=\frac{1}{T}\sum_{\mu ,v=t,b}j_{\mu }^{T}\rho_{\mu ,v}j_{v}+\frac{1}{T^{2}}(\nabla_{x}T{)}^{T}\kappa_{j}\nabla_{x}T,$$

where $\kappa_{j}\equiv \kappa_{E}-T\sum_{\mu =t,b}\tau_{\mu }^{T}\alpha_{\mu }$ is the thermal conductivity under zero normal current, in which *κ_**ε**_* ≡ ∑_*μ=t,b*_ *κ_μ_* is the thermal conductivity under an electrochemical potential gradient of zero.

## III. EXCITON CONDENSATION AND SUPERFLUIDITY

Now that the formalism for coupled double layer systems has been established in such a way that includes exciton formation, one may introduce exciton condensation, which occurs at sufficiently low temperature and whose environment can support superfluidity. The exciton condensate can be described by the condensate wave function $\Psi (x,\tau )=\sqrt{n_{e}^{c}(x,\tau )}\text{exp}(i\theta (x,\tau ))$. The exciton condensate velocity is given by

(30) $$v_{e}^{c}(x,\tau )=\frac{\hbar }{m_{e}}\nabla_{x}\theta (x,\tau ),$$

where *m_e_* is the exciton effective mass. At finite temperature, the collision integral between exciton condensate and noncondensate excitons, given in Appendix A, will make the transport coefficients implicitly depend on the local condensate density $n_{e}^{c}$ and momentum $m_{e}v_{e}^{c}$. The hydrodynamic equations for the condensate can be written as [17]

(31) $$\frac{\partial n_{e}^{c}}{\partial \tau }+\nabla_{x}\cdot \left(n_{e}^{c}v_{e}^{c}\right)=-\int C_{e}^{c}[f](k_{e})dk_{e},\;\;m_{e}\frac{\partial v_{e}^{c}}{\partial \tau }=-\nabla_{x}\left(\zeta_{c}+\frac{m_{e}{\left(v_{e}^{c}\right)}^{2}}{2}\right),$$

where the chemical potential of an exciton condensate is [17,18]

(32) $$\zeta_{c}=-\frac{\nabla_{x}^{2}\sqrt{n_{e}^{c}}}{2m_{e}\sqrt{n_{e}^{c}}}+V_{ex}+gn_{e}^{c}+2gn_{e}^{n}$$

in which *g* is the strength of the *s*-wave approximated interexciton potential, *V_ex_* is the external potential on excitons such that ∇_**x**_*V_ex_* = **F**_*e*_, and $n_{e}^{n}=\int f_{e}(k_{e})dk_{e}$ is the density of the noncondensate excitons.

At thermal equilibrium, we expect zero current for the exciton condensate $\left(v_{e}^{c}=0\right)$, otherwise, there is a nonzero noncondensate exciton current that can generate finite entropy production. When small external forces or temperature gradients are applied, as seen in previous sections, a proportionate response is expected by the transport currents, or average velocity of noncondensate excitons and unbounded carriers. These driving forces will also induce a nonzero velocity of exciton condensate, similar to the case of an induced supercurrent in superconductors due to a temperature gradient [49,50]. To lowest order, we can assume $v_{e}^{c}$ is proportional to the external force or temperature gradient, and we can safely neglect the term $\frac{m_{e}(v_{e}^{c}{)}^{2}}{2}$ in Eq. (31) and the quantum pressure term in *ζ_c_*. Therefore, to have a stationary flow of exciton condensate, the chemical potential *ζ_c_* must be position independent, from which the exciton condensate density $n_{e}^{c}$ can be determined.

We can decompose the total electric current in layer *μ* as

(33) $$J_{\mu }=j_{\mu }^{n}+(q_{t}\delta_{\mu t}+q_{b}\delta_{\mu b})j_{e}^{s},$$

where $j_{e}^{s}=n_{e}^{c}v_{e}^{c}$ is the supercurrent of excitons and $j_{\mu }^{n}$ is the normal component of the electric current in layer *μ* given by Eq. (12). The exciton condensate velocity $v_{e}^{c}$ can be determined from the continuity equation in Eq. (31), which is

(34) $$\nabla_{x}\cdot j_{e}^{s}=-\int C_{e}^{c}[f](k_{e})dk_{e}=\sum_{\mu =t,b,e}A_{\mu }\left(v_{e}^{c}\right)\cdot F_{\mu }-B(v_{e}^{c})\cdot \nabla_{x}T$$

where

(35) $$A_{\mu }(v_{e}^{c})\equiv \int Q_{e}^{c}(k_{e}^{\prime }k_{e}^{\prime \prime };k_{e}k_{e}^{c})G_{\mu }(k_{e}k_{e}^{\prime }k_{e}^{\prime \prime })dk_{e}dk_{e}^{\prime }dk_{e}^{\prime \prime },\;\;B(v_{e}^{c})\equiv Q_{e}^{c}(k_{e}^{\prime }k_{e}^{\prime \prime };k_{e}k_{e}^{c})H(k_{e}k_{e}^{\prime }k_{e}^{\prime \prime })dk_{e}dk_{e}^{\prime }dk_{e}^{\prime \prime },$$

with $\hbar k_{e}^{c}=m_{e}v_{e}^{c}$ being the momentum of the condensate, shorthand notation $G_{\mu }(k_{e}k_{e}^{\prime }k_{e}^{\prime \prime })$ for $G_{\mu }(k_{e})-G_{\mu }(k_{e}^{\prime })-G_{\mu }(k_{e}^{\prime \prime })$ and $H(k_{e}k_{e}^{\prime }k_{e}^{\prime \prime })$ for $H(k_{e})-H(k_{e}^{\prime })-H(k_{e}^{\prime \prime })$, and

(36) $$G_{\mu }(k_{e})=L_{e,\mu }(\nabla_{k}n_{\mu }),\;\;H(k_{e})=\sum_{\mu =t,b,e}L_{e,\mu }\left(\frac{e_{\mu }-\zeta_{\mu }}{T}\nabla_{k}n_{\mu }\right).$$

It is worth noting that the energy spectrum of the noncondensed excitons or unbounded charge carriers can be altered by the presence of the exciton condensation. The mathematical notation for all of these descriptions will be consistent throughout this paper.

The right hand side of Eq. (34) may be nonzero since the external force and temperature gradient can be varied arbitrarily, indicating that the conversion between condensate and noncondensate is due to the interaction between them. It can be shown that $A_{\mu }(v_{e}^{c}=0)=B(v_{e}^{c}=0)=0$, so that both sides of Eq. (34) are of second order about $F_{\mu }$ and ∇_**x**_*T*. In fact, the implicit conservation laws encoded in $Q_{e}^{c}$ in Eq. (35) are

(37) $$k_{e}^{c}+k_{e}=k_{e}^{\prime }+k_{e}^{\prime \prime },\;e_{e}(k_{e})+\zeta_{c}+\frac{{\left(v_{e}^{c}\right)}^{2}}{2m_{e}}=e_{e}(k_{e}^{\prime })+e_{e}(k_{e}^{\prime }).$$

If $k_{e}^{c}=0$, Eq. (37) has inversion symmetry and thus both processes of $k_{e}↔k_{e}^{\prime }+k_{e}^{\prime \prime }$ and $-k_{e}↔-k_{e}^{\prime }-k_{e}^{\prime \prime }$ have equal probability; that is, $Q_{e}^{c}(k_{e}^{\prime }k_{e}^{\prime \prime };k_{e},k_{e}^{c}=0)$ remains the same when the direction of **k**_*e*_, $k_{e}^{\prime }$, and $k_{e}^{\prime \prime }$ are simultaneously reversed. Since both **G**_*μ*_ (**k**_*e*_) and **H**(**k**_*e*_) are odd functions, $A_{\mu }(v_{e}^{c})$ and $B(v_{e}^{c})$ must be zero when $v_{e}^{c}=0$. We can thus expand $A_{\mu }(v_{e}^{c})$ and $B(v_{e}^{c})$ around $v_{e}^{c}=0$ and rewrite Eq. (34) as

(38) $$\nabla_{x}\cdot j_{e}^{s}=v_{e}^{c}\cdot \left[\sum_{\mu =t,b,e}(\nabla_{v}⊗A_{\mu })F_{\mu }-(\nabla_{v}⊗B)\nabla_{x}T\right],$$

where the differential operator ∇_**v**_ is applied on $A_{\mu }(v_{e}^{c})$ and $B(v_{e}^{c})$ with respect to $v_{e}^{c}$, and ∇_**v**_ ⊗ **A**_*μ*_ and ∇_**v**_ ⊗ **B** are evaluated at $v_{e}^{c}=0$. The right hand side of Eq. (38) is of second order. We can then safely consider $j_{e}^{s}$ as divergenceless. On the other hand, any global scaling of $v_{e}^{c}$ leaves Eq. (38) invariant. We can then denote $v_{e}^{c}=v{\hat{v}}_{e}^{c}$, where ${\hat{v}}_{e}^{c}$ is a dimensionless solution of Eq. (38).

Now *v* can be fixed by invoking the principle of minimum entropy production [51,52]. Since the exciton condensate carries no entropy, the local entropy production in Eq. (29) can be rewritten as

(39) s=1T[∑μ,v=t,bJμTρμ,vJv+jesTWjes][−2∑μ=t,bjesT(qtρt,μ+qbρb,μ)Jμ]+1T2(∇xT)Tκj∇xT

where

(40) $$W\equiv q_{t}^{2}\rho_{t,t}+q_{t}q_{b}\rho_{t,b}+q_{b}q_{t}\rho_{b,t}+q_{b}^{2}\rho_{b,b}.$$

Note that the term $\int (e_{e}-\zeta_{e})C_{e}^{c}[f](k_{e})dk_{e}∼v^{3}$ is neglected and not added to Eq. (28). Therefore, the existence of exciton condensation will not produce entropy; instead, it redistributes entropy among excitations.

The total entropy production is

(41) S=∫s(x)dx,

where the area integration is over the whole plane. Minimum entropy production then requires that *∂S/∂v* = 0. The systems under consideration have uniform flows and thus uniform entropy production in the interior of the system. The contribution to the total entropy production from the boundary of the system can be neglected. The minimum total entropy production is then considered to be equivalent to the local minimum entropy production, that is $\frac{\partial s}{\partial v}=0$. Neglecting the second order terms, we can then obtain

(42) $$v=\frac{\sum_{\mu =t,b}{\left({\hat{v}}_{e}^{c}\right)}^{T}(q_{t}\rho_{t,\mu }+q_{b}\rho_{b,\mu })J_{\mu }}{n_{e}^{c}{\left({\hat{v}}_{e}^{c}\right)}^{T}W{\hat{v}}_{e}^{c}}$$

and the normal current

(43) $$j_{\mu }^{n}=J_{\mu }-\frac{q_{t}\delta_{t\mu }+q_{b}\delta_{b\mu }}{{\left({\hat{v}}_{e}^{c}\right)}^{T}W{\hat{v}}_{e}^{c}}\sum_{v=t,b}\left({\hat{v}}_{e}^{c}⊗{\hat{v}}_{e}^{c}\right)(q_{t}\rho_{t,v}+q_{b}\rho_{b,v})J_{v}.$$

Now, Eq. (12) can be written as

(44) $$E_{\mu }-\alpha_{\mu }\nabla_{x}T=\sum_{v}{\tilde{\rho }}_{\mu ,v}J_{v},$$

where the new resistivity matrix $\tilde{\rho }$ is

(45) $${\tilde{\rho }}_{\mu ,v}=\rho_{\mu ,v}-\frac{\left(q_{t}\rho_{\mu ,t}+q_{b}\rho_{\mu ,b})\left({\hat{v}}_{e}^{c}⊗{\hat{v}}_{e}^{c}\right)(q_{t}\rho_{t,v}+q_{b}\rho_{b,v}\right)}{{\left({\hat{v}}_{e}^{c}\right)}^{T}W{\hat{v}}_{e}^{c}}.$$

It can be verified that the Onsager relation holds, i.e., ${\tilde{\rho }}_{\mu ,v}^{T}={\tilde{\rho }}_{v,\mu }$.

The matrix composed of blocks of ${\tilde{\rho }}_{\mu ,v}$ is singular because $j_{\mu }^{n}=0$ if $J_{\mu }=(q_{t}\delta_{t\mu }+q_{b}\delta_{b\mu })u{\hat{v}}_{e}^{c}$ for any given *u* below the critical value; that is, all the current will be carried by the supercurrent. Therefore, the presence of exciton condensation will yield a singular resistivity matrix and the system can support supercurrent and generate zero entropy production from carrier transport.

There are no explicit modifications for the other coefficients in Eqs. (15) and (12). However, those coefficients are implicitly affected by the existence of exciton condensate and its interaction with noncondensate excitons.

## IV. THERMOELECTRIC APPLICATIONS

It can be shown that the heat current in each layer can be rewritten as

(46) $$h_{\mu }=\sum_{v,\omega =t,b}\chi_{\mu ,\omega }q_{\omega }\rho_{\omega ,v}j_{v}^{n}+\left(\sum_{v=t,b}\chi_{\mu ,v}q_{v}\alpha_{v}-\kappa_{\mu }\right)\nabla_{x}T$$

and the total heat current is

(47) $$h=\sum_{\mu =t,b}h_{\mu }=\sum_{\mu =t,b}T\alpha_{\mu }^{T}j_{\mu }^{n}-\kappa_{j}\nabla_{x}T.$$

The first term in Eq. (47) can be identified with the Peltier term. When all the electric current is carried by the exciton condensate, the Peltier term vanishes and we have **h** = −*κ*_**j**_∇_**x**_*T*. Then the input and the output heat current cancels, resulting in no energy production and contradicting the situation of a finite Seebeck voltage that can drive an external load. In these cases, the energy balancing at the contact interfaces plays an important role to resolve such contradictions.

**FIG. 3.**
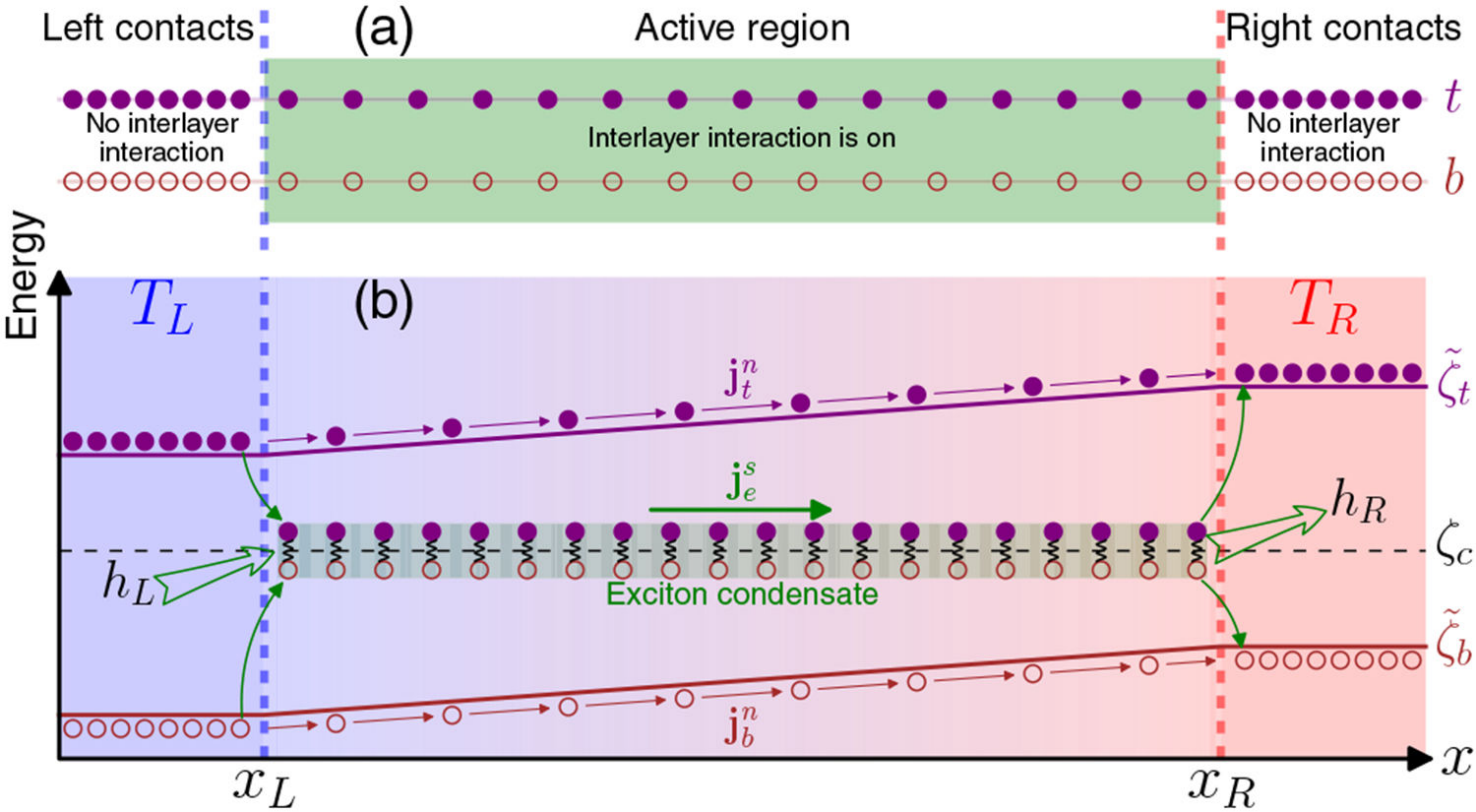
Schematic (a) of the double layer system with contacts and an active region having a green background. Its energy diagram is shown in (b) while in the presence of a temperature gradient. The purple solid dots and red circles represent unbounded carriers in each layer. The layers are labeled by *t* and *b* at their right ends in (a). The line labels for electrochemical potentials ${\tilde{\zeta }}_{t}$ and ${\tilde{\zeta }}_{b}$ and chemical potential *ζ_c_* of exciton condensate are labeled at right end of each line in (b). The vertical dashed lines are the interfaces between the active region and the contacts that are in thermal equilibrium with thermal reservoirs at temperatures *T_L_* and *T_R_*. The heat current *h_L_* (*h_R_*) flowing from (into) the left (right) contact is indicated by the outlined green arrow. The exciton condensate is in the green region in (b). Normal currents $j_{t}^{n}$ and $j_{b}^{n}$ and exciton condensate current $j_{e}^{s}$ are also labeled. The thin, curved green arrows represent the processes of exciton formation, dissociation, or condensation. The signs of the gradient of ${\tilde{\zeta }}_{t}$ and ${\tilde{\zeta }}_{b}$ in the active region are positive in this illustration.

Considering a system that is uniform in one direction, the transport is essentially reduced to 1D, as in the schematics in Fig. 3. The vector quantities then become scalars. However, the bold-style conventions are kept for them (e.g., **j** and **h** are actually scalars). In Fig. 3(a), the system is composed of a double layer with left contacts, an active region, and right contacts, separated by the vertical blue and red dashed interfacial lines. The interlayer Coulomb interaction is on only in the active regions so that there are no excitons or exciton condensation in the contacts. The left and right contacts are in thermal equilibrium with two reservoirs at temperatures of *T_L_* and *T_R_*, respectively. Near the interfaces, excitons are formed and condensed, or uncondensed and dissociated, depending on the direction of the exciton current. Any excitonic reactions happen within the healing length of an exciton condensate, accompanied by an energy release for exciton formation and absorption for exciton dissociation. The electrochemical potential ${\tilde{\zeta }}_{\mu }$ for each layer is drawn in Fig. 3(b) as well as the flat chemical potential *ζ_c_* of exciton condensate. The electrochemical potential is related to its gradient by

(48) $$E_{\mu }=-\frac{1}{q_{\mu }}\nabla_{x}{\tilde{\zeta }}_{\mu }$$

for *μ* = *t, b* and $F_{e}=-\nabla_{x}{\tilde{\zeta }}_{e}$ where ${\tilde{\zeta }}_{e}={\tilde{\zeta }}_{t}+{\tilde{\zeta }}_{b}$. We now use the energy conservation law at the interfacial regions:

(49) $$dU=\tilde{\zeta }dN+T\;dS+E_{r}dN,$$

where *U* is the internal energy, $\tilde{\zeta }$ is the electrochemical potential for unbounded carriers or chemical potential for excitons or exciton condensate, *N* is the number of carriers, *E_r_* is the reaction energy (e.g., binding energy *E_r_* = *E_b_*) for exciton formation/dissociation/condensation, and *S* is the entropy. The term *T* d*S* can be identified with the heat exchange.

At steady state, we have d*U*/d*t* = 0. Therefore, for the left interface (blue dashed line in Fig. 3), we have

(50) hL+∣(jes(ζ~t+ζ~b−ζc)−h+jenEb+jesEbc)∣xL=0,

where *E_b_* is the exciton binding energy, *E_bc_* is the exciton condensation energy, *h_L_* is the heat flowing out of the left contact, and $j_{e}^{n}$ is the normal exciton current that can be written as

(51) $$j_{e}^{n}=\int v_{e}(k_{e})f_{e}dk_{e}=\sum_{v=t,b}\sigma_{e,v}F_{v}-\tau_{e}\nabla_{x}T,$$

with

(52) $$\sigma_{e,v}=\frac{k_{B}T}{\hbar^{2}}(J_{e,e}+J_{e,v}),\;\;\tau_{e}=\frac{k_{B}}{\hbar^{2}}(K_{e,e}+K_{e,t}+K_{e,b}).$$

Similarly for the right interface

(53) $${(j_{e}^{s}(\zeta_{c}-{\tilde{\zeta }}_{t}-{\tilde{\zeta }}_{b})+h-j_{e}^{n}E_{b}-j_{e}^{s}E_{bc})|}_{x_{R}}-h_{R}=0.$$

In order to simplify the analysis, we assume that both the transport coefficients and the energies *E_b_* and *E_bc_* have negligible temperature dependence. At thermal equilibrium, we have $\zeta_{c}={\tilde{\zeta }}_{e}={\tilde{\zeta }}_{t}+{\tilde{\zeta }}_{b}$ in which all terms are independent of position. When a temperature gradient is applied, the electrochemical potentials are no longer flat. Instead, ${\tilde{\zeta }}_{e}$ will intersect with the flat ${\tilde{\zeta }}_{c}$ at the center of the active region, indicating a symmetrical distribution of the magnitude of nonequilibrium exciton concentrations at both interfaces. Thus we can write

(54) $$({\tilde{\zeta }}_{t}+{\tilde{\zeta }}_{b}-\zeta_{c}){|}_{x_{L}}=-({\tilde{\zeta }}_{t}+{\tilde{\zeta }}_{b}-\zeta_{c}){|}_{x_{R}}\;\;=\frac{1}{2}[{\tilde{\zeta }}_{t}(x_{L})-{\tilde{\zeta }}_{t}(x_{R})+{\tilde{\zeta }}_{b}(x_{L})-{\tilde{\zeta }}_{b}(x_{R})],$$

in which

(55) $${\tilde{\zeta }}_{\mu }(x_{L})-{\tilde{\zeta }}_{\mu }(x_{R})\;\;=-q_{\mu }\left[\alpha_{\mu }(T_{L}-T_{R})-l\sum_{v}{\tilde{\rho }}_{\mu ,v}J_{v}\right],$$

where *l* = *x_R_* − *x_L_* is the length of the active region.

Now we can obtain the heat current *h_L_* and *h_R_*:

(56) $$h_{L}=\frac{j_{e}^{s}}{2}(q_{t}\alpha_{t}+q_{b}\alpha_{b})(T_{L}-T_{R})+T_{L}\sum_{\mu =t,b}\alpha_{\mu }j_{\mu }^{n}\;\;-\kappa_{j}\frac{T_{R}-T_{L}}{l}-\frac{l}{2}q_{J}-j_{e}^{n}E_{b}-j_{e}^{s}E_{bc},\;h_{R}=\frac{j_{e}^{s}}{2}(q_{t}\alpha_{t}+q_{b}\alpha_{b})(T_{R}-T_{L})+T_{R}\sum_{\mu =t,b}\alpha_{\mu }j_{\mu }^{n}\;\;-\kappa_{j}\frac{T_{R}-T_{L}}{l}+\frac{l}{2}q_{J}-j_{e}^{n}E_{b}-j_{e}^{s}E_{bc},$$

where $q_{J}\equiv \sum_{\mu ,v=t,b}j_{\mu }^{n}\rho_{\mu ,v}j_{v}^{n}$ is the Joule heating density inside the active region. Note that the equality $q_{t}{\tilde{\rho }}_{t,v}+q_{b}{\tilde{\rho }}_{b,v}=0$ has been used. Now the energy production is

(57) $$h_{L}-h_{R}=(T_{L}-T_{R})\sum_{\mu =t,b}\alpha_{\mu }J_{\mu }-lq_{J}.$$

The first term on the right hand side of Eq. (57) is readily identified as the thermoelectric power generation and it must be positive.

The thermoelectric power generation requires that the entropy production rate of the two thermal reservoirs is positive, i.e.,

(58) $$\sigma_{R}=\frac{h_{R}}{T_{R}}-\frac{h_{L}}{T_{L}}>0.$$

Three typical cases are summarized below, and although other general cases can be studied in a similar fashion, their derivations tend to be too tedious to document here.

### A. No excitons or exciton condensation

When there are no interlayer excitons, the formulations above may be reduced to the traditional analysis of thermoelectric transport, with interlayer Coulomb drag resistivity included. To simply the formula without losing the effect of the interlayer Coulomb interaction, we assume that *α_b_* = −*α_t_* = *α*, *ρ_t,t_* = *ρ_b,b_* = *ρ*_0_, *ρ_t,b_* = *ρ_b,t_* = *ρ_D_*, and *T_R_* = *T_h_* > *T_L_* = *T_c_*. It can be shown that the counterflow current satisfies

(59) $$I_{CF}=J_{t}=-J_{b}=\frac{\alpha (T_{h}-T_{c})}{R_{x}+l(\rho_{0}-\rho_{D})},$$

where the load resistance is 2*R_x_* in the counterflow configuration shown in Fig. 1. The thermoelectric efficiency can now be written as

(60) $$\eta =\eta_{C}\frac{m}{l+m}{\left(1+\frac{l+m}{ZT_{h}l}-\frac{\eta_{C}}{2}\frac{l}{l+m}\right)}^{-1},$$

where $\eta_{C}=\frac{T_{h}-T_{c}}{T_{h}}$ is the Carnot efficiency, *m* = *R_x_*/(*ρ*_0_ − *ρ_D_*), and *ZT_h_* is the figure of merit

(61) $$ZT_{h}=Z_{0}T_{h}\frac{1}{1-\rho_{D}∕\rho_{0}},$$

where $Z_{0}T_{h}=\frac{2\alpha^{2}}{\kappa_{j}\rho_{0}}T_{h}$. Clearly, positive *ρ_D_* is beneficial for enhancing the *ZT_h_* value if the other transport coefficients are not significantly affected by the interlayer Coulomb interaction. The *ZT_h_* value can be dramatically enhanced if there is strong Coulomb drag such that the Coulomb drag resistivity *ρ_D_* is very close to the intralayer resistivity *ρ*_0_. The formula for *η* under generic resistivity matrix and Seebeck coefficient values can be similarly obtained but have explicit forms that are too lengthy to express here. Now we see that the effective conductance in Eq. (4) and *ZT* formula of Eq. (5) deduced from the phenomenological model may not be appropriate for predicting thermoelectric efficiency, but they are still useful to demonstrate the importance of interlayer interactions for enhancing thermoelectric efficiency in double layer systems.

### B. Only excitons without exciton condensation

When the concentration of the unbounded carriers in the active region is low and neglected, all the currents are carried by the noncondensed excitons when both *T_L_* and *T_R_* are above the transition temperature of exciton condensation. The resistivity matrix is singular and thus the Seebeck coefficients for individual layers are not well defined. Instead, we can write the Seebeck coefficient for an exciton as

(62) $$\alpha_{e}=q_{t}\alpha_{t}+q_{b}\alpha_{b}=\rho_{e}\tau_{e}=\frac{K_{e,e}}{J_{e,e}},$$

where $\rho_{e}\equiv \sigma_{e,e}^{-1}$, so that the heat currents are

(63) $$h_{L}=T_{L}\alpha_{e}j_{e}^{n}-\kappa_{j}\frac{T_{R}-T_{L}}{l}-\frac{l}{2}q_{J}-j_{e}^{n}E_{b},\;h_{R}=T_{R}\alpha_{e}j_{e}^{n}-\kappa_{j}\frac{T_{R}-T_{L}}{l}+\frac{l}{2}q_{J}-j_{e}^{n}E_{b},$$

where $q_{J}=\rho_{e}(j_{e}^{n}{)}^{2}$. The exciton current is

(64) $$j_{e}^{n}=\frac{(T_{L}-T_{R})\alpha_{e}}{l\rho_{e}+q^{2}R_{x}},$$

where *q* = *q_t_* = −*q_b_* and we again assume a load resistance of *R_x_* in the counterflow configuration shown in Fig. 1. We also assume *T_R_* = *T_h_* > *T_L_* = *T_c_*. The thermoelectric efficiency can then be written as

(65) $$\eta \equiv \frac{h_{L}-h_{R}}{-h_{R}}=\eta_{C}\frac{1-\frac{l}{l+m}}{1+\frac{1}{ZT_{h}}\frac{l+m}{l}-\frac{\eta_{C}}{2}\frac{l}{l+m}-\frac{E_{b}}{\alpha_{e}T_{h}}},$$

where *m* = *q*^2^*R_x_*/*ρ_e_* and

(66) $$ZT_{h}=\frac{\alpha_{e}^{2}}{\kappa_{j}\rho_{e}}T_{h}=\frac{\alpha_{e}^{2}\sigma_{e,e}}{\kappa_{j}}T_{h}.$$

Equation (65) can be recast into Eq. (60) if we define an effective figure of merit as

(67) $$Z_{\text{eff}}T_{h}\equiv \frac{ZT_{h}}{1-ZT_{h}\frac{E_{b}}{\alpha_{e}T_{h}}\frac{l}{l+m}}.$$

The entropy production rate can be written as

(68) $$\sigma_{R}=\frac{(T_{h}-T_{c}{)}^{2}}{T_{h}T_{c}}\left[\frac{\alpha_{e}^{2}(T_{h}+T_{c})l}{2\rho_{e}(l+m{)}^{2}}-\frac{E_{b}\alpha_{e}}{\rho_{e}(l+m)}+\frac{\kappa_{j}}{l}\right].$$

Positive entropy production requires that

(69) $$\frac{E_{b}}{\alpha_{e}T_{h}}<\frac{l+m}{ZT_{h}l}+\left(1-\frac{\eta_{C}}{2}\right)\frac{l}{l+m}.$$

**FIG. 4.**
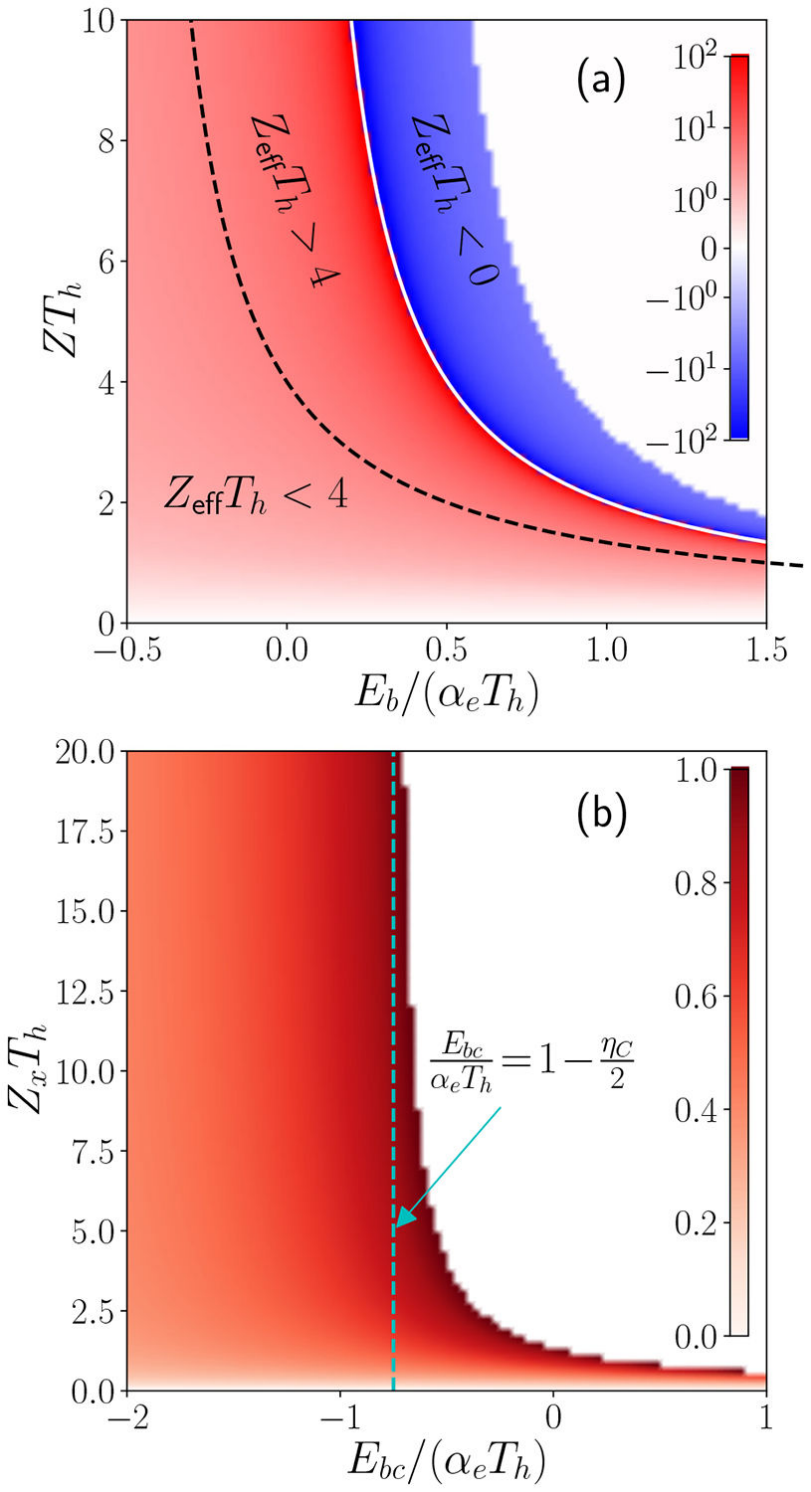
Color maps of (a) *Z*_eff_*T_h_* in Eq. (67) and (b) *η/η_C_* in Eq. (72), where $Z_{x}T_{h}\equiv \frac{\alpha_{e}^{2}T_{h}}{\kappa_{j}(2q^{2}R_{x}∕l)}$ is the extrinsic thermoelectric figure of merit.

The color map of *Z*_eff_*T_h_* is shown in Fig. 4(a), where we use representative values of $\eta_{C}=\frac{l}{l+m}=0.5$. Note that *Z*_eff_*T_h_* can be divergent (on the white line) and even negative (blue region). We have *Z*_eff_*T_h_* > 4 in the red region between the dashed line and the white line. Thermoelectric power generation is not allowed in the blank area as the entropy production is negative.

### C. Excitons with exciton condensation

When both *T_R_* and *T_L_* are below the transition temperature of exciton condensation and the concentration of unbounded carriers is small enough to be neglected, the current in the active region is carried entirely by the exciton condensate, thus *q_J_* = 0. The heat currents are

(70) $$h_{L}=\frac{j_{e}^{s}}{2}\alpha_{e}(T_{L}-T_{R})-\kappa_{j}\frac{T_{R}-T_{L}}{l}-j_{e}^{s}E_{bc},\;h_{R}=\frac{j_{e}^{s}}{2}\alpha_{e}(T_{R}-T_{L})-\kappa_{j}\frac{T_{R}-T_{L}}{l}-j_{e}^{s}E_{bc},$$

It has been predicted that interlayer voltages do not allow stationary exciton condensate flow [53,54]. Therefore, the serial counterflow connection cannot be used in thermoelectric applications in the presence of exciton condensation. Instead, each layer needs to form an individual current loop. The exciton condensate current is

(71) $$j_{e}^{s}=\frac{(T_{L}-T_{R})\alpha_{e}}{2q^{2}R_{x}}$$

when a load resistance *R_x_* is connected to each layer to form two individual loops. We use the same convention that *T_R_* = *T_h_* > *T_L_* = *T_c_*. The thermoelectric efficiency is

(72) $$\eta \equiv \frac{h_{L}-h_{R}}{-h_{R}}=\eta_{C}\frac{1}{\frac{\eta_{C}}{2}+\kappa_{j}\frac{2q^{2}R_{x}}{l\alpha_{e}^{2}T_{h}}-\frac{E_{bc}}{\alpha_{e}T_{h}}},$$

and the entropy production rate is

(73) $$\sigma_{R}=\frac{(T_{h}-T_{c}{)}^{2}}{T_{h}T_{c}}\left[\frac{\kappa_{j}}{l}-\frac{\alpha_{e}^{2}(T_{h}+T_{c})}{4q^{2}R_{x}}-\frac{E_{bc}\alpha_{e}}{2q^{2}R_{x}}\right].$$

Note that the traditionally defined figure of merit term is absent in Eq. (72) because of the absence of exciton resistivity *ρ_e_*. Instead, we can define an extrinsic figure of merit

(74) $$Z_{x}T_{h}\equiv \frac{\alpha_{e}^{2}T_{h}}{\kappa_{j}(2q^{2}R_{x}∕l)},$$

since it depends on the load resistance 2*R_x_* and system length *l*. The color map of *η/η_C_* is shown in Fig. 4(b) (a representative value of *η_C_* = 0.5 is used). The vertical dashed line defines the bound of $\frac{E_{bc}}{\alpha_{e}T_{h}}$ for either a long active region (*l* → ∞) or a small load resistance (*R_x_* → 0), under which Eq. (72) is reduced to

(75) $$\eta =\eta_{C}{\left(\frac{\eta_{C}}{2}-\frac{E_{bc}}{\alpha_{e}T_{h}}\right)}^{-1}.$$

For this case, in order to guarantee positive *σ_R_*, we need

(76) $$0<-\alpha_{e}<\frac{2E_{bc}}{T_{h}+T_{c}},$$

which requires $j_{e}^{s}>0$. In other words, exciton condensation current flows from cold to hot, resembling the superfluid fountain effect. Letting $\frac{T_{h}+T_{c}}{2}$ be close to the Berezinskii-Kosterlitz-Thouless (BKT) transition temperature [55], i.e.,

(77) $$\frac{T_{h}+T_{c}}{2}\approx T_{\text{BKT}}\approx \frac{0.078}{2\pi }\frac{E_{bc}}{k_{B}},$$

the relation $-\alpha_{e}\approx \frac{2E_{bc}}{T_{h}+T_{c}}$ then requires $|\frac{\alpha_{e}}{q}|\approx 1.6$ mV/K, which is large, yet possibly attainable in Bi-based double layer exciton systems [42].

## V. DISCUSSION AND CONCLUDING REMARKS

It has been established that the role of exciton condensates in the enhancement of thermoelectric efficiency is twofold: (1) Exciton condensates redistribute the contribution of electrical and heat current in the system in such a way that electrical current is carried by the exciton condensate, whereas the heat current is carried by exciton excitations and other excitations. This different functionality provides a new way of designing thermoelectric modules. Mathematically, the existence of exciton condensate can increase the effective electrical conductivity to infinity, thus extending the traditional ZT to infinity as well. However, a complete thermoelectric system must have interfaces and external loads, preventing an actual ‘infinite’ case to emerge. (2) In the case of coupled double layer systems, the lateral dimensions contribute to the thermoelectric efficiency formula in Eq. (72), thus making length parameters another degree of consideration in device fabrication.

As shown in Eqs. (65) and (72), the exciton binding and condensation energies are required in order to calculate thermoelectric efficiency, in addition to the traditional thermoelectric figure of merit. Even for a double layer system without interlayer excitons, the figure of merit is modified so that the Coulomb drag resistivity enters the thermoelectric figure of merit of Eq. (61). Therefore, bringing double layers close enough at the nanometer scale can dramatically enhance thermoelectric efficiency in a way that cannot be realized in more traditional approaches where the conflicting materials’ parameters limit the enhancement.

The interaction between layers leading to high Coulomb drag resistivity or interlayer exciton formation can be controllably tuned, adding a powerful new ingredient to engineer the thermoelectric efficiency and power output. We can have efficiencies close to Carnot efficiency when excitons or exciton condensation are present, given the appropriate conditions. Though exciton condensation bears significant physical similarities with conventional superconductors and BEC of helium or dilute Bose gases [56,57], their thermoelectric responses differ fundamentally.

The theoretical inquiries presented herein have predicted a phenomenon, similar to the superfluid fountain effect [58,59], whereby exciton condensation current flows from cold to hot areas. The latter fountain effect is due to the conversion from superfluid to normal and takes place at the heated spots, resulting in a superfluid flow toward those heated spots. Traditionally, one expects a constriction (such as thin tubes) to slow down or stop the flow of the normal fluid component in order to exhibit the fountain effect. Such constrictions are subtle to realize in double layer systems for exciton condensate. In fact, for an exciton condensate realized in bilayer quantum wells in the quantum Hall regime, though the parallel transport is very resistive, the counterflow transport that supports exciton flow can be very conductive [60]. A larger phase space has been explored for exciton condensation in bilayer graphene double layer systems [8,9], however no thermoelectric transport measurement has been performed.

In superconductors, no thermoelectric voltage can be detected in a uniform superconductor due to the effortless compensation of the normal thermoelectric current by an opposite supercurrent [49]. A nonzero thermoelectric response can only be observed in an inhomogeneous superconductor, e.g., bimetallic superconducting loop [50]. In Ref. [50], a similar minimization procedure is carried out to obtain the supercurrent component of the circulating current in the bimetallic loop and the results satisfactorily agreed with the experiments. Though finite supercurrents can be induced with a temperature gradient in superconductor loops, a superconductor might not be useful for generating thermoelectric power. Significant thermoelectric voltage can only be generated in a small range around the interfaces between superconducting and normal metal or between two superconductors, whereas a bulk superconductor has exponentially small thermoelectric voltage due to its energy gap. Thus, superconductors as thermoelectric channeling materials cannot effectively drive an external load, since there are only insignificant temperature drops in those small ranges that are usually at microscopic scales. These drawbacks worsen when the length of the superconductor is macroscopically large.

Unlike Cooper pairs, excitons are charge neutral and thus exciton supercurrent cannot carry an electric current by itself, but exciton supercurrent contributes to the counterflow of electric currents in both layers of the double layer system. Though the individual layer electrochemical potentials need to satisfy the requirement of ${\tilde{\zeta }}_{e}={\tilde{\zeta }}_{t}+{\tilde{\zeta }}_{b}$, ${\tilde{\zeta }}_{t}$ and ${\tilde{\zeta }}_{b}$ can have appreciable gradients because the electric current and carrier densities in each individual layer can be tuned by external circuits. However, for Cooper pairs in superconductors, both electrons in the pair are in the same environment, lacking the degree of freedom of layer identities for excitons.

An exciton binding energy can be large due to the tunable nature and strength of interlayer Coulomb coupling, permitting high temperature exciton condensate superfluidity [55]. This enables thermoelectric applications within a large temperature range. Even if there is no exciton condensation [55], the noncondensed exciton gas and large exciton binding energy can facilitate entering the regimes in Fig. 4(a) where the effective *Z*_eff_*T* is larger than 4 or even negative, thus supporting once again that coupled double layer systems offer unique opportunities to realize high efficiency thermoelectric modules. One interesting direction that can be taken with this formalism is to explore the temperature dependence of the thermoelectric efficiency given the conditions described in the previous sections.

To conclude, quantum Boltzmann transport formalism was used to study the thermoelectric response of coupled double layer systems. The interaction between exciton condensate and noncondensate is included using the Zaremba-Nikuni-Griffin formalism [17,18]. We show that the Onsager relation holds for the resistivity matrix even when an exciton condensation exists, and the resistivity matrix is singular as expected. We then use the obtained transport coefficients to derive the formulas for thermoelectric efficiency when the coupled double layer system is used as the active region connecting two thermal reservoirs. It is crucial to take the energy of exciton formation, dissociation, and condensation into account, allowing one to resolve the dilemma that exciton condensation flow can carry electric current but no entropy. This mathematical demonstration clarifies the route for engineering systems to enhance thermoelectric efficiency.

## Appendix

### APPENDIX A: COLLISION INTEGRALS

The collision integrals are defined as

(A1) Cμs[f](x,kμ)=∫[S1(x∣kμ′;kμ)fμ′f¯μ−S1(x∣kμ;kμ′)fμf¯μ′]dkμ′,Cμb[f](x,kμ)=∑v∫[S2(x∣kμ′kv′;kμkv)fμ′fv′f¯μf¯v−S2(x∣kμkv;kμ′kv′)fμfvf¯μ′f¯v′]dkμ′dkvdkv′,Cμr[f](x,kμ)=∑α,β∫{12[S3(x∣kαkβ;kμ)fαfβf¯μ−S3(x∣kμ;kαkβ)fμf¯αf¯β]}{+S3(x∣kα;kμkβ)fαf¯μf¯β−S3(x∣kμkβ;kα)fμfβf¯α}dkαdkβ,Cec[f](ke)=∫[Sc(ke′ke″;kekec)f¯efe′fe″−Sc(kekec;ke′ke″)fef¯e′f¯e″][+2Sc(ke″kec;keke′)f¯ef¯e′f¯e″−2Sc(keke′;ke″kec)fef¯e′f¯e″]dke′dke″,

where $\hbar k_{e}^{c}=m_{e}v_{e}^{c}$ is the momentum of the exciton condensate (*m_e_* is the mass of excitons). The transition probability *S_c_* is proportional to the condensate density.

We have used the shorthand notation of $f_{\mu }^{\prime }=f_{\mu }(x,k_{\mu }^{\prime },t)$. We have defined ${\bar{f}}_{\mu }\equiv 1-(-1{)}^{\delta_{\mu e}}f_{\mu }$ since the unbounded carriers and excitons are considered to be fermions and bosons, respectively, where *δ_μe_* is the Kronecker delta function. These collision integrals describe the single particle scatterings (phonons, defects, impurities, etc.), particle-particle scatterings, exciton formation and dissociation, and scattering between the condensate and noncondensate [17,18,44], with their corresponding transition rates *S*_1_, *S*_2_, *S*_3_, and *S_c_*, respectively. For example, $S_{1}(x|k_{\mu }^{\prime };k_{\mu })$ is the transition probability of the single particle scattering from state with momentum $k_{\mu }^{\prime }$ to state with momentum **k**_*μ*_, *S*_3_(**x**∣**k***_t_***k***_b_*; **k***_e_*) is the probability of the formation of the exciton with quasimomentum **k***_e_* from particles with quasimomentum **k***_t_* and **k***_b_*. Note that in the integral *C_μ_*, $k_{v}^{\prime }$ needs to be replaced by $k_{\mu }^{\prime \prime }$, when *ν* = *μ*. The factor $\frac{1}{2}$ in $C_{\mu }^{r}$ comes from the fact that *S*_3_(**x**∣**k**_*α*_**k**_*β*_; **k**_*μ*_) is symmetrical with respect to the exchange of *α* and *β*. It is also understood that *α* = *t* and *β* = *b* or *α* = *b* and *β* = *t* in *S*_3_(**x**∣**k**_*α*_**k**_*β*_; **k**_*μ*_) for *μ* = *e*; otherwise *S*_3_ is zero. The conservation laws (energy, momentum, charge, etc.) and the quantum mechanical nature of the scattering is implicitly encoded in the mathematical forms of the transition probabilities. For example, exciton formation/dissociation requires

(A2) $$k_{t}+k_{b}=k_{e}+G_{e},$$

where **G**_*e*_ is some reciprocal lattice vector corresponding to the lattice composed of all the translation vectors **R**_*e*_ that leave the total Hamiltonian invariant when the translation is applied simultaneously to both layers.

When external electrical fields **E**_*t/b*_ are applied to each layer, Eq. (6) needs to be supplemented by the following equations of semiclassical dynamics:

(A3) $$\partial_{\tau }k_{\mu }=\frac{1}{\hbar }F_{\mu },\;\;\partial_{\tau }x=v_{\mu }(k_{\mu })=\frac{1}{\hbar }\nabla_{k}e_{\mu }(k_{\mu }),$$

where **F**_*μ*_ = *q_μ_***E**_*μ*_ is the force on the unbounded carriers carrying charge *q_μ_* in the layer *μ* (= *t, b*), **F**_*e*_ = **F**_*t*_ + **F**_*b*_ is the force on the excitons, *e_μ_*(**k**_*μ*_) and $v_{\mu }(k_{\mu })=\frac{1}{\hbar }\nabla_{k}e_{\mu }(k_{\mu })$ are the band structure and group velocity of unbounded carriers (*μ* = *t, b*) and noncondensed excitons (*μ* = *e*).

### APPENDIX B: LINEARIZING THE COLLISION INTEGRALS

The linearized collision integrals are

(B1) $$C_{\mu }^{s}[\phi ](k_{\mu })=\int Q_{1}(k_{\mu }^{\prime };k_{\mu })(\phi_{\mu }-\phi_{\mu }^{\prime })dk_{\mu }^{\prime },\;C_{\mu }^{b}[\phi ](k_{\mu })=\sum_{v}\int Q_{2}(k_{\mu }^{\prime }k_{v}^{\prime };k_{\mu }k_{v})(\phi_{\mu }+\phi_{v}-\phi_{\mu }^{\prime }-\phi_{v}^{\prime })dk_{\mu }^{\prime }dk_{v}dk_{v}^{\prime },\;C_{\mu }^{r}[\phi ](k_{\mu })=\sum_{\alpha ,\beta }\int \left[\frac{1}{2}Q_{3}(k_{\alpha }k_{\beta };k_{\mu })(\phi_{\mu }-\phi_{\alpha }-\phi_{\beta })+Q_{3}(k_{\mu }k_{\beta };k_{\alpha })(\phi_{\mu }+\phi_{\beta }-\phi_{\alpha })\right]dk_{\alpha }dk_{\beta },\;C_{e}^{c}[\phi ](k_{e})=\int \left[Q_{c}(k_{e}^{\prime }k_{e}^{\prime \prime };k_{e}k_{e}^{c})(\phi_{e}-\phi_{e}^{\prime }-\phi_{e}^{\prime \prime })+2Q_{c}(k_{e}^{\prime \prime }k_{e}^{c};k_{e}k_{e}^{\prime })(\phi_{e}+\phi_{e}^{\prime }-\phi_{e}^{\prime \prime })\right]dk_{e}^{\prime }dk_{e}^{\prime \prime },$$

where

(B2) $$Q_{1}(k_{\mu }^{\prime };k_{\mu })\equiv S_{1}(k_{\mu }^{\prime };k_{\mu })n_{\mu }^{\prime }{\bar{n}}_{\mu },\;\;Q_{2}(k_{\mu }^{\prime }k_{v}^{\prime };k_{\mu }k_{v})\equiv S_{2}(k_{\mu }^{\prime }k_{v}^{\prime };k_{\mu }k_{v})n_{\mu }^{\prime }n_{v}^{\prime }{\bar{n}}_{\mu }{\bar{n}}_{v},\;Q_{3}(k_{\alpha }k_{\beta };k_{\mu })\equiv S_{3}(k_{\alpha }k_{\beta };k_{\mu })n_{\alpha }n_{\beta }{\bar{n}}_{\mu },\;\;Q_{c}(k_{e}^{\prime }k_{e}^{\prime \prime };k_{e}k_{e}^{c})\equiv S_{c}(k_{e}^{\prime }k_{e}^{\prime \prime };k_{e}k_{e}^{c}){\bar{n}}_{e}n_{e}^{\prime }n_{e}^{\prime \prime }.$$

The principle of detailed balance requires

(B3) $$Q_{1}(k_{\mu }^{\prime };k_{\mu })=Q_{1}(k_{\mu };k_{\mu }^{\prime }),\;\;Q_{3}(k_{\alpha }k_{\beta };k_{\mu })=Q_{3}(k_{\mu };k_{\alpha }k_{\beta })\equiv S_{3}(k_{\mu };k_{\alpha }k_{\beta })n_{\mu }{\bar{n}}_{\alpha }{\bar{n}}_{\beta },\;Q_{2}(k_{\mu }^{\prime }k_{v}^{\prime };k_{\mu }k_{v})=Q_{2}(k_{\mu }k_{v};k_{\mu }^{\prime }k_{v}^{\prime }),\;\;Q_{c}(k_{e}^{\prime }k_{e}^{\prime \prime };k_{e}k_{e}^{c})=Q_{c}(k_{e}k_{e}^{c};k_{e}^{\prime }k_{e}^{\prime \prime })\equiv S_{c}(k_{e}k_{e}^{c};k_{e}^{\prime }k_{e}^{\prime \prime })n_{e}{\bar{n}}_{e}^{\prime }{\bar{n}}_{e}^{\prime \prime }.$$

Additional symmetries are

(B4) $$Q_{2}(k_{\mu }^{\prime }k_{v}^{\prime };k_{\mu }k_{v})=Q_{2}(k_{v}^{\prime }k_{\mu }^{\prime };k_{v}k_{\mu }),\;\;Q_{3}(k_{\alpha }k_{\beta };k_{\mu })=Q_{3}(k_{\beta }k_{\alpha };k_{\mu }).$$

By defining the linear operator $P_{\mu ,v}=P_{\mu ,v}^{s}+P_{\mu ,v}^{b}+P_{\mu ,v}^{r}+P_{\mu ,v}^{c}$ with

(B5) $$P_{\mu ,v}^{s}\phi_{v}\equiv \int Q_{1}(k_{\mu }^{\prime };k_{\mu })(\phi_{\mu }-\phi_{\mu }^{\prime })dk_{\mu }^{\prime }\delta_{\mu v},\;P_{\mu ,v}^{b}\phi_{v}\equiv \sum_{\alpha }\int Q_{2}(k_{\mu }^{\prime }k_{\alpha }^{\prime };k_{\mu }k_{\alpha })(\phi_{\mu }-\phi_{\mu }^{\prime })dk_{\mu }^{\prime }dk_{\alpha }dk_{\alpha }^{\prime }\delta_{\mu v}\int Q_{2}(k_{\mu }^{\prime }k_{v}^{\prime };k_{\mu }k_{v})(\phi_{v}-\phi_{v}^{\prime })dk_{\mu }^{\prime }dk_{v}dk_{v}^{\prime },\;P_{\mu ,v}^{r}\phi_{v}\equiv \sum_{\alpha ,\beta }\int \left[\frac{1}{2}Q_{3}(k_{\alpha }k_{\beta };k_{\mu })+Q_{3}(k_{\mu }k_{\beta };k_{\alpha })\right]\phi_{\mu }dk_{\alpha }dk_{\beta }\delta_{\mu v}\;\;+\sum_{\alpha }\int [Q_{3}(k_{\mu }k_{v};k_{\alpha })-Q_{3}(k_{\mu }k_{\alpha };k_{v})-Q_{3}(k_{v}k_{\alpha };k_{\mu })]\phi_{v}dk_{\alpha }dk_{v},\;P_{e,e}^{c}\phi_{e}=Q_{c}^{a}(k_{e}k_{e}^{c})\phi_{e}+\int Q_{c}^{b}(k_{e}k_{e}^{c};k_{e}^{\prime })\phi_{e}^{\prime }dk_{e}^{\prime },$$

where

(B6) $$Q_{c}^{a}(k_{e}k_{e}^{c})\equiv \int [Q_{c}(k_{e}^{\prime }k_{e}^{\prime \prime };k_{e}k_{e}^{c})+2Q_{c}(k_{e}^{\prime \prime }k_{e}^{c};k_{e}k_{e}^{\prime })]dk_{e}^{\prime }dk_{e}^{\prime \prime }$$

and

(B7) $$Q_{c}^{b}(k_{e}k_{e}^{c};k_{e}^{\prime })\equiv \int [2Q_{c}(k_{e}^{\prime \prime }k_{e}^{c};k_{e}k_{e}^{\prime })-2Q_{c}(k_{e}^{\prime }k_{e}^{\prime \prime };k_{e}k_{e}^{c})-2Q_{c}(k_{e}^{\prime }k_{e}^{c};k_{e}k_{e}^{\prime \prime })]dk_{e}^{\prime }dk_{e}^{\prime \prime },$$

the collision integral now becomes

(B8) $$C_{\mu }[\phi ](k_{\mu })=\sum_{v}P_{\mu ,v}\phi_{v}.$$

Generalization of the above derivation is made for the generic collision integral that has explicit and simple physical significance which can be written as

(B9) $$C_{\mu }^{g}[f]=\sum_{\begin{array}{c} \alpha \ldots \\ \beta \ldots \end{array}}\int [S(k_{\alpha }\ldots ;k_{\beta }\ldots )f_{\alpha }\ldots {\bar{f}}_{\beta }\ldots -S(k_{\beta }\ldots ;k_{\alpha }\ldots )f_{\beta }\ldots {\bar{f}}_{\alpha }\ldots ]dk_{\alpha }\ldots dk_{\beta }\ldots ,$$

where one of the *f*s for the second part of the arguments (**k**_*β*_ …) in *S*(**k**_*α*_ …; **k**_*β*_ …), e.g., *f_β_* is identified with *f_μ_* by including a Dirac delta *δ*(*μβ*) in the transition probabilities *S*(…;…). The collision integral $C_{\mu }^{g}[f]$ represents the processes that the incoming states with momentum **k**_*α*_ … are scattered into the outgoing states with momentum **k**_*β*_…. The linearized $C_{\mu }^{g}[f]$ can be written as

(B10) $$C_{\mu }^{g}[\phi ]=\sum_{\begin{array}{c} \alpha \ldots \\ \beta \ldots \end{array}}\int Q(k_{\alpha }\ldots ;k_{\beta }\ldots )(\phi_{\beta }+\cdots -\phi_{\alpha }-\cdots )dk_{\beta }\ldots dk_{\alpha }\ldots ,$$

where

(B11) $$Q(k_{\alpha }\ldots ;k_{\beta }\ldots )\equiv S(k_{\alpha }\ldots ;k_{\beta }\ldots )n_{\alpha }\ldots {\bar{n}}_{\beta }\ldots ,\;\;Q([\rho \sigma ]\ldots ;[\alpha \beta ]\ldots )=Q([\alpha \beta ]\ldots ;[\rho \sigma ]\ldots ),$$

due to detailed balance. Without losing the generality, we can assume that *f*_*β*_ is identified with *f*_*μν*_, i.e.,

(B12) $$C_{\mu }^{g}[\phi ]=\sum_{\begin{array}{c} \alpha \ldots \\ v\ldots \end{array}}\int Q(k_{\alpha }\ldots ;k_{\mu }k_{v}\ldots )(\phi_{\mu }+\phi_{v}+\cdots -\phi_{\alpha }-\cdots )dk_{v}\ldots dk_{\alpha }\ldots ,$$

so that we have

(B13) $$Q^{a}(k_{\mu })=\sum_{\begin{array}{c} \alpha \ldots \\ v\ldots \end{array}}\int Q(k_{\alpha }\ldots ;k_{\mu }k_{v}\ldots )dk_{v}\ldots dk_{\alpha }\ldots$$

and

(B14) $$Q^{b}(k_{\mu };k_{\nu })=\sum_{\begin{array}{c} \ldots \alpha \ldots \end{array}}\int [Q(\ldots k_{\alpha }\ldots ;k_{\mu }\ldots k_{\nu }\ldots )-Q(\ldots k_{v}\ldots ;k_{\mu }\ldots k_{\alpha }\ldots )]dk_{\alpha }\ldots ,$$

where the summation in *Q^b^* excludes the index *ν* and similarly for the integral elements d**k**_*ν*_. It can be verified that *Q^a^* and *Q^b^* satisfy Eqs. (21) and (22).

### APPENDIX C: ELECTRIC AND HEAT CURRENT

The electric current in each layer (*μ* = *t, b*) is

(C1) $$j_{\mu }^{n}=q_{\mu }\sum_{v=\mu ,e}\int v_{v}(k_{v})f_{v}dk_{v}=-q_{\mu }\sum_{v=\mu ,e}\int_{B_{v}}v_{v}(k_{v})n_{v}{\bar{n}}_{v}\phi_{v}dk_{v}\;\;=\frac{k_{B}Tq_{\mu }}{\hbar^{2}}\sum_{\begin{array}{c} v=\mu ,e\\ \alpha =t,b,e \end{array}}\int \nabla_{k}n_{v}L_{v,\alpha }\left[\left(F_{\alpha }-\frac{e_{\alpha }-\zeta_{\alpha }}{T}\nabla_{x}T\right)\cdot \nabla_{k}n_{\alpha }\right]dk_{v}\equiv j_{\mu }^{F}+j_{\mu }^{T},$$

where

(C2) $$j_{\mu }^{F}=\frac{k_{B}Tq_{\mu }}{\hbar^{2}}\sum_{\begin{array}{c} v=\mu ,e\\ \alpha =t,b,e \end{array}}\left[\int \nabla_{k}n_{v}⊗L_{v,\alpha }\nabla_{k}n_{\alpha }dk_{v}\right]F_{\alpha },\;j_{\mu }^{T}=-\frac{k_{B}q_{\mu }}{\hbar^{2}}\sum_{\begin{array}{c} v=\mu ,e\\ \alpha =t,b,e \end{array}}\left[\int \nabla_{k}n_{v}⊗L_{v,\alpha }[(e_{\alpha }-\zeta_{\alpha })\nabla_{k}n_{\alpha }]dk_{v}\right]\nabla_{x}T,$$

and we have used the notation (**a** ⊗ **b**)_*mn*_ ≡ *a_m_b_n_*. We can define

$$J_{\mu ,v}\equiv \int \nabla_{k}n_{\mu }⊗L_{\mu ,v}\nabla_{k}n_{v}dk_{\mu }.$$

We then have

(C3) $$j_{\mu }^{F}=\frac{k_{B}Tq_{\mu }}{\hbar }\sum_{v=t,b}(J_{\mu ,v}+J_{\mu ,e}+J_{e,v}+J_{e,e})F_{v}.$$

The heat current in each layer is defined as

(C4) $$h_{\mu }=\sum_{v=\mu ,e}\int v_{v}(k_{v})\frac{e_{v}(k_{v})-\zeta_{v}}{1+\delta_{ve}}f_{v}dk_{v}$$

and its calculation is straightforward.
